# Increased Risk of Alzheimer Disease–Associated Mortality in Nonobese vs Obese Metabolic Dysfunction–Associated Steatotic Liver Disease: A 30-Year National Cohort Study

**DOI:** 10.1016/j.gastha.2026.101030

**Published:** 2026-06-09

**Authors:** Sarpong Boateng, Guy Loic Nguefang, Mexan Mapouka, Ridwan Lawal, Solomon Gyabaah, Bright Nwatamole, Amita Kasar, Selom Adatsi, Basile Njei

**Affiliations:** 1 Section of Digestive Diseases, Yale University, New Haven, Connecticut; 2 Department of Medicine, Yale New Haven Health, Bridgeport Hospital, Bridgeport, Connecticut; 3 College of Public Health, University of North Texas, Fort Worth, Texas; 4 Department of Medicine, Texas Tech University Health Sciences Center, Lubbock, Texas; 5 Department of Medicine, University of Maryland Capital Region Medical Center, Largo, Maryland; 6 Department of Medicine, University of Texas Rio Grande Valley, Edinburg, Texas; 7 Department of Medicine, Komfo Anokye Teaching Hospital, Kumasi, Ghana; 8 Department of Medicine, Zucker School of Medicine at Hofstra/Northwell at Vassar Brothers Medical Center, Poughkeepsie, New York; 9 Department of Medicine, Virginia Mason Medical Center, Seattle, Washington; 10 Department of Medicine, Ryazan State Medical University, Ryazan, Russia

**Keywords:** Alzheimer Disease, Metabolic Dysfunction–Associated Steatotic Liver Disease, Mortality, Obese

## Abstract

**Background and Aims:**

Metabolic dysfunction–associated steatotic liver disease (MASLD) is increasingly recognized for its extrahepatic consequences, including emerging links to neurodegenerative disorders such as Alzheimer disease (AD). Whether AD mortality risk differs across MASLD phenotypes, remains unclear.

**Methods:**

We analyzed adults from the Third National Health and Nutrition Examination Survey (1988–1994) with mortality follow-up through 2019 via the National Death Index. Participants were followed for AD mortality. Cumulative incidence was estimated using Kaplan–Meier methods. Cox proportional hazards models evaluated MASLD phenotypes and AD mortality, adjusting for age, sex, race/ethnicity, poverty-income ratio, body mass index, and smoking status.

**Results:**

Among 7125 adults, 1033 had nonobese MASLD and 817 had obese MASLD. At baseline, nonobese MASLD participants were older (mean age 60 ± 12 years), more likely male (59%), and more frequently White (46%) compared with obese MASLD (mean age 56 ± 13 years, 46% male, 37% White; *P* < .001). Age-standardized cumulative incidence of AD mortality was highest in nonobese MASLD (1.98%), followed by non-MASLD (1.81%) and obese MASLD (0.78%). In adjusted models, MASLD was not significantly associated with AD mortality overall. However, nonobese MASLD was independently associated with higher AD mortality compared with obese MASLD (adjusted hazard ratio 3.76; 95% confidence interval 1.19, 11.90; *P* = .024) and with the overall population (adjusted hazard ratio 1.49; 95% confidence interval 1.03, 2.16; *P* = .034).

**Conclusion:**

Nonobese MASLD emerged as distinct high-risk metabolic phenotype associated with significantly higher AD mortality, independent of demographic, socioeconomic, and behavioral factors. These findings suggest that nonobese MASLD may reflect unique neuro-metabolic vulnerability and warrant further mechanistic investigation into pathways such as differential adiposity patterns, inflammation, and metabolic signaling. Targeted screening, improved risk stratification, and prospective studies are needed to better define and mitigate long-term cognitive risks in this understudied subgroup.

## Introduction

Metabolic dysfunction–associated steatotic liver disease (MASLD), previously termed nonalcoholic fatty liver disease, refers to a heterogeneous group of liver disorders that arise in the setting of metabolic dysregulation, particularly insulin resistance. The condition is defined by abnormal hepatic lipid deposition occurring in individuals who consume little to no alcohol.^1^ MASLD has emerged as the most common cause of chronic liver disease worldwide and frequently coexists with key features of metabolic syndrome, such as excess adiposity, type 2 diabetes mellitus, dyslipidemia, and elevated blood pressure. Central to its pathogenesis are sustained inflammatory pathways and oxidative injury, supporting the concept of MASLD as a multisystem disease with clinically relevant effects beyond the liver, including an increased risk of extrahepatic comorbidities.^2^, ^3^, ^4^, ^5^

Alzheimer disease (AD) is a chronic, progressive neurodegenerative condition and the leading cause of dementia in older adults, characterized by gradual cognitive decline and loss of functional independence. The burden of AD continues to expand globally in parallel with population aging, creating a significant and growing strain on health-care systems.^6^ While its pathogenesis is complex and influenced by an interplay among genetic susceptibility, environmental exposures, and lifestyle factors, increasing evidence suggests a potential role for metabolic dysfunction in the development and progression of the disease.^7^

The link between MASLD and AD is supported by shared pathophysiological pathways, which include insulin resistance, chronic low-grade inflammation, oxidative stress, dyslipidemia, and endothelial and microvascular dysfunction. These mechanisms are implicated in amyloid-β accumulation, tau pathology, and cerebrovascular injury. Such overlapping mechanisms underpin a liver-brain axis, whereby hepatic metabolic dysfunction may contribute to neurodegenerative risk and disease progression.^8^

Epidemiologic research has increasingly examined the relationship between steatotic liver disease and cognitive outcomes across various dementia types.^9^^,^^10^ Multiple prospective cohort studies have identified links between MASLD and the development of dementia, with stronger connections often seen in vascular dementia cases. However, findings related to AD have been varied and inconsistent.^10^^,^^11^ Moreover, most earlier research focused on incident dementia diagnosis rather than cause-specific mortality, leaving the long-term impact of MASLD on AD-related death largely unexplored.^10^^,^^11^

A critical knowledge gap exists concerning the heterogeneity of MASLD across the body mass index (BMI) spectrum and its various associations with neurologic outcomes. Nonobese MASLD is increasingly acknowledged as a distinct clinical and biological phenotype, frequently characterized by adverse metabolic profiles, increased sarcopenia, and heightened systemic inflammation, despite the absence of obesity.^8^^,^^9^ In parallel, an “obesity paradox” has been observed in late-life cognitive outcomes and dementia-related mortality, where higher BMI or stable weight may be linked to a lower risk of dementia. This could reflect metabolic reserve, survivor bias, or reverse causation from weight loss before the onset of clinical dementia.^12^^,^^13^ The extent to which these phenomena influence differential AD-related mortality outcomes between individuals with nonobese vs obese MASLD remains inadequately understood within a long-term prospective national cohort.^10^^,^^11^ To address this deficiency, we analyzed the national health survey to assess AD-associated mortality among individuals with MASLD, stratified by obesity status. We hypothesize that nonobese MASLD is independently associated with increased AD-related mortality compared to obese MASLD, even after adjusting for established cardiometabolic risk factors. An improved understanding of long-term neurologic mortality risks across various MASLD phenotypes could enhance risk stratification, facilitate the identification of high-risk groups, and inform personalized preventive strategies in the rapidly expanding population of patients with MASLD.

## Methods

### Study Design and Population

We conducted a prospective cohort analysis using data from the Third National Health and Nutrition Examination Survey (NHANES III; 1988–1994), a nationally representative survey of the noninstitutionalized US civilian population conducted by the National Center for Health Statistics using a complex, multistage, stratified probability sampling design. NHANES III combined standardized interviews, physical examinations, and laboratory assessments, with oversampling of older adults and racial/ethnic minority groups to enhance population representativeness.

Adults aged ≥20 years who completed the mobile examination center evaluation, had interpretable hepatic ultrasonography and were eligible for mortality linkage were included. Participants were followed from the date of baseline examination until death or December 31, 2019, whichever occurred first.

To isolate MASLD, we excluded individuals with competing causes of hepatic steatosis or chronic liver disease, including significant alcohol consumption (>21 drinks/week in men or >14 drinks/week in women), hepatitis B surface antigen positivity, hepatitis C antibody positivity, evidence of iron overload (transferrin saturation >50%), ungradable ultrasound images, or missing key exposure, covariate, or mortality follow-up data.

### Assessment of Hepatic Steatosis and Metabolic Dysfunction–Associated Steatotic Liver Disease Phenotypes

Hepatic steatosis was assessed using standardized gallbladder-focused ultrasonography performed during the NHANES III examination and later centrally reviewed by trained radiologists following a validated protocol. Steatosis was graded based on liver echogenicity, hepatorenal contrast, vessel wall definition, and parenchymal attenuation. Hepatic steatosis was categorized as none, mild, moderate, or severe, and only participants with moderate or severe steatosis were classified as having MASLD to enhance diagnostic specificity, consistent with prior NHANES III–based mortality studies.

MASLD phenotypes were defined according to BMI using ethnicity-specific thresholds. Nonobese MASLD was defined as MASLD with BMI <30 kg/m^2^ in non-Asian participants and <25 kg/m^2^ in Asian participants. Obese MASLD was defined as MASLD with BMI at or above these thresholds. BMI was calculated from measured height and weight obtained during the mobile examination center examination.

In sensitivity analyses, lean MASLD was defined using stricter cutoffs (BMI <25 kg/m^2^ for non-Asian participants and <23 kg/m^2^ for Asian participants) to assess the robustness of associations across alternative adiposity definitions.

### Mortality Ascertainment and Outcome Definition

Vital status and cause of death were ascertained through linkage with the NHANES III Linked Mortality File, conducted by the National Center for Health Statistics of the Centers for Disease Control and Prevention using probabilistic matching to the National Death Index. The linkage algorithm incorporates multiple identifiers, including Social Security number, name, date of birth, sex, race, and state of residence and has demonstrated a match confirmation rate exceeding 98% in validation studies. Mortality follow-up is publicly available and described in detail by the Centers for Disease Control and Prevention (https://www.cdc.gov/nchs/data-linkage/mortality.htm).

The primary outcome was AD–specific mortality, defined as AD listed as the underlying cause of death on death certificates (International Classification of Diseases, Ninth Revision [ICD-9] code 331.0 and International Classification of Diseases, Tenth Revision [ICD-10] codes G30.x). Participants who died from non-AD causes were censored at the time of death. Follow-up time was calculated in months and converted to years for time-to-event analyses.

### Covariates

Covariates were selected a priori based on established associations with MASLD, neurodegenerative disease, and mortality, while avoiding adjustment for downstream mediators. Demographic variables included age, sex, and race/ethnicity. Social and geographic factors included marital status, US Census region, and rural vs metropolitan residence. Socioeconomic status was assessed using the poverty-income ratio.

Socioeconomic status was assessed using the poverty-income ratio (<1 [below poverty], 1–<3, ≥3). Smoking status was categorized as never, former, or current smoker based on lifetime cigarette use and current smoking behavior. Physical activity was defined based on self-reported activity relative to peers (less active, about the same, more active). Social engagement was assessed by asking whether participants reported participating in clubs or organizations (yes/no). BMI was included as a continuous variable in multivariable models to account for residual confounding beyond phenotype classification.

### Statistical Analysis

Baseline characteristics were summarized using survey-weighted means and proportions. Group differences were assessed using Kruskal–Wallis tests for continuous variables and χ^2^ tests for categorical variables, accounting for the complex survey design.

The cumulative incidence of AD mortality was estimated using the Kaplan–Meier method. Age-standardized cumulative incidence was calculated using the 2000 US Census population as the reference. Survival curves were compared across MASLD phenotypes using log-rank tests.

Survey-weighted Cox proportional hazards models were used to estimate adjusted hazard ratios (aHRs) and 95% confidence intervals (CIs) for the association between MASLD phenotypes and AD mortality. Primary contrasts included MASLD vs non-MASLD, nonobese MASLD vs obese MASLD, and nonobese MASLD vs all other participants. Proportional hazards assumptions were evaluated using Schoenfeld residuals and log–log survival plots, with no violations observed.

Sensitivity analyses evaluated alternative BMI-based definitions of lean MASLD and compared lean MASLD with nonlean MASLD and with the overall population. All analyses incorporated NHANES III sampling weights, strata, and primary sampling units to ensure nationally representative estimates and appropriate variance estimation. Statistical significance was defined as a two-sided *P* value <.05. Analyses were performed using R (version 4.5.1) and SAS (version 9.4).

### Ethical Consideration

As all data are aggregated and deidentified, institutional review board approval was not required. The North Texas regional institutional review board (IRB) determined this project, involving strictly publicly available datasets, does not meet the definition of human subject research under the purview of the IRB according to federal regulations. Therefore, IRB review of this project was not required.

## Results

### Study Population

A total of 7125 adults from NHANES III met the inclusion criteria and were followed through December 31, 2019. Of these, 5275 (74.0%) did not have MASLD, 817 (11.5%) had obese MASLD, and 1033 (14.5%) had nonobese MASLD. Median follow-up exceeded 25 years, providing long-term ascertainment of AD-specific mortality.

### Baseline Characteristics

Baseline characteristics stratified by MASLD phenotype are shown in Table 1. Participants with nonobese MASLD were older at baseline (mean age 60 ± 12 years) compared with those with obese MASLD (56 ± 13 years) and non-MASLD participants (65 ± 17 years; *P* < .001). Nonobese MASLD participants were more likely to be male (59%) and non-Hispanic White (46%) compared with obese MASLD (46% male; 37% White), *P* < .001.

**Table 1:** Baseline Characteristics of NHANES III Adults by MASLD Phenotype

Characteristic^a^	Non-MASLD N = 5275	Obese MASLD N = 817	Nonobese MASLD N = 1033	*P* value^b^
Age (y), mean (SD)	65 (17)	56 (13)	60 (12)	<.001
Sex, N (%)				<.001
Male	2671 (51%)	372 (46%)	609 (59%)	
Female	2604 (49%)	445 (54%)	424 (41%)	
Race/ethnicity				<.001
NH White	2905 (55%)	301 (37%)	477 (46%)	
NH Black	1379 (26%)	219 (27%)	241 (23%)	
Mexican American	854 (16%)	271 (33%)	290 (28%)	
Other	137 (2.6%)	26 (3.2%)	25 (2.4%)	
Region				<.001
Northeast	791 (15%)	105 (13%)	136 (13%)	
Midwest	1151 (22%)	156 (19%)	181 (18%)	
South	2337 (44%)	358 (44%)	504 (49%)	
West	996 (19%)	198 (24%)	212 (21%)	
Marital status				<.001
Married/partnered	2965 (56%)	498 (61%)	698 (68%)	
Widowed	1328 (25%)	120 (15%)	139 (13%)	
Divorced/separated	542 (10%)	123 (15%)	130 (13%)	
Never married	429 (8.1%)	74 (9.1%)	63 (6.1%)	
Rural/urban				.20
Metro	2276 (43%)	353 (43%)	413 (40%)	
Nonmetro	2999 (57%)	464 (57%)	620 (60%)	
Poverty-income ratio, N (%)				.003
<1 (below poverty)	1076 (20%)	205 (25%)	199 (19%)	
1-3	2276 (43%)	361 (44%)	461 (45%)	
≥3	1923 (36%)	251 (31%)	373 (36%)	
Body mass index (kg/m^2^), mean (SD)	25.7 (4.8)	35.1 (4.8)	25.7 (2.9)	<.001
Smoking status, N (%)				<.001
Never smoker	2257 (43%)	348 (43%)	353 (34%)	
Former smoker	1697 (32%)	280 (34%)	393 (38%)	
Current smoker	1321 (25%)	189 (23%)	287 (28%)	
Physical activity vs peers, N (%)				<.001
About the same	2159 (42%)	351 (43%)	476 (47%)	
Less active than peers	1075 (21%)	278 (34%)	207 (21%)	
More active than peers	1894 (37%)	178 (22%)	324 (32%)	
Club/organization membership (yes), N (%)	1710 (32%)	241 (29%)	346 (33%)	.20

NH, non-Hispanic.

a Mean (SD); n (%).

b Kruskal-Wallis rank sum test; Pearson Chi-square test.

Significant differences were also observed across geographic region, marital status, poverty–income ratio, smoking status, and physical activity (all *P* < .001). Participants with obese MASLD were more likely to report lower physical activity compared with peers (34%) than those with nonobese MASLD (21%) and non-MASLD (21%). In contrast, nonobese MASLD participants were more frequently married or partnered (68%) compared with obese MASLD (61%) and non-MASLD participants (56%). Rural vs urban residence (metro: 40%–43%; *P* = .20) and club or organizational membership (29%–33%; *P* = .20) did not differ significantly across MASLD phenotypes (Table 1).

### Cumulative Incidence of Alzheimer Disease Mortality

During follow-up, 196 AD-specific deaths occurred. Crude cumulative incidence of AD mortality was highest among participants with nonobese MASLD (3.78%), followed by non-MASLD participants (2.77%) and obese MASLD participants (1.35%) (Table 2).

**Table 2:** Cumulative Incidence of Alzheimer Disease Deaths in NHANES III Adults by MASLD Phenotype

Group	N	AD deaths	Crude CI	Standardized CI
Non-MASLD	5275	146	2.77%	1.81%
Obese MASLD	817	11	1.35%	0.78%
Nonobese MASLD	1033	39	3.78%	1.98%

CI, cumulative incidence.

After age standardization to the 2000 US Census population, nonobese MASLD remained associated with the highest cumulative incidence of AD mortality (1.98%), compared with non-MASLD (1.81%) and obese MASLD (0.78%).

### Survival Analyses

**Figure 1:**
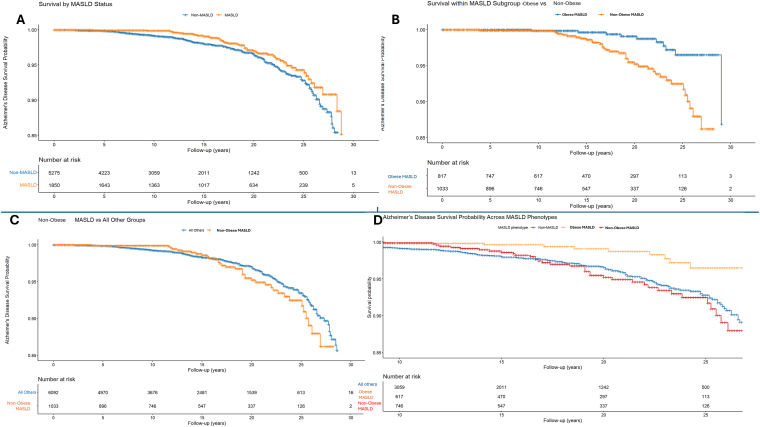
Kaplan–Meier curves for Alzheimer disease–specific survival according to metabolic dysfunction–associated steatotic liver disease (MASLD) phenotype in NHANES III participants followed through 2019. (A) Alzheimer disease–specific survival among participants with MASLD vs those without MASLD. (B) Alzheimer disease–specific survival among participants with MASLD stratified by obesity status (obese MASLD vs nonobese MASLD). (C) Alzheimer disease–specific survival comparing nonobese MASLD with all other participants combined (obese MASLD and non-MASLD). (D) Alzheimer disease–specific survival across all 3 phenotypes simultaneously (non-MASLD, obese MASLD, and nonobese MASLD).

Kaplan–Meier survival curves demonstrated differential AD-free survival across MASLD phenotypes (Figure 1). Overall survival did not differ significantly between participants with MASLD and those without MASLD (Panel A). However, within the MASLD subgroup, participants with nonobese MASLD experienced significantly lower AD-free survival compared with those with obese MASLD (Panel B).

Compared with all other participants combined, nonobese MASLD demonstrated a persistently lower survival probability throughout follow-up (Panel C). In a three-group comparison, nonobese MASLD exhibited the lowest AD-free survival, followed by non-MASLD, while obese MASLD showed the highest survival probability (Panel D).

### Multivariable Cox Proportional Hazards Models

In fully adjusted Cox proportional hazards models, MASLD overall was not associated with AD mortality compared with non-MASLD (aHR 1.17; 95% CI 0.81, 1.67; *P* = .40).

In contrast, nonobese MASLD was independently associated with a substantially higher risk of AD mortality compared with obese MASLD (aHR 3.76; 95% CI 1.19, 11.90; *P* = .024). Nonobese MASLD was also associated with higher AD mortality when compared with all other participants (aHR 1.49; 95% CI 1.03, 2.16; *P* = .034) (Table 3).

**Table 3:** Adjusted Hazard Ratios for Alzheimer Disease Mortality by MASLD Phenotype

Exposure	Adjusted HR^a^	95% CI	*P* value
MASLD vs no MASLD	1.17	0.81–1.67	.40
Nonobese MASLD vs obese MASLD	3.76	1.19–11.90	.024
Nonobese MASLD vs all other participants	1.49	1.03–2.16	.034
Sensitivity analyses			
Lean MASLD vs nonlean MASLD^b^	1.87	1.02–3.45	.044
Lean MASLD vs all other participants^b^	1.79	1.07–3.00	.027

a Adjusted for age, sex, race/ethnicity, marital status, region, rural/urban status, poverty-income ratio, BMI, physical activity, smoking status, and social interaction.

b Lean MASLD, defined as MASLD with BMI <23 kg/m^2^ in Asian participants and <25 kg/m^2^ in non-Asian participants.

### Sensitivity Analyses

Using a stricter definition of lean MASLD (BMI <25 kg/m^2^ in non-Asian participants and <23 kg/m^2^ in Asian participants), results were consistent with the primary analysis. Lean MASLD was associated with increased AD mortality compared with nonlean MASLD (aHR 1.87; 95% CI 1.02, 3.45; *P* = .044) and compared with all other participants (aHR 1.79; 95% CI 1.07, 3.00; *P* = .027).

Across all analyses, proportional hazards assumptions were met, and effect estimates were consistent in direction and magnitude.

## Discussion

In this nationally representative cohort of US adults with more than 3 decades of follow-up, we demonstrate that AD mortality risk varies substantially by MASLD phenotype. While MASLD overall was not associated with AD mortality, nonobese MASLD emerged as a distinct high-risk phenotype with significantly higher AD-specific mortality compared with obese MASLD and with the overall population. These associations were robust across extensive multivariable adjustment and persisted in sensitivity analyses using stricter definitions of lean MASLD. Collectively, our findings highlight significant heterogeneity within MASLD and identify nonobese disease as a previously underrecognized neurodegenerative risk state.

### Novelty in the Context of Prior East Asian Studies

Prior evidence linking hepatic steatosis phenotypes to dementia outcomes has been mainly derived from East Asian populations, particularly Korean cohorts, where lean or nonobese fatty liver disease is more prevalent and metabolic profiles differ substantially from those of Western populations.^14^^,^^15^ These studies have suggested an association between lean nonalcoholic fatty liver disease and incident dementia; however, their generalizability to the US population has remained uncertain due to several key limitations.^14^ East Asian cohorts are comparatively homogeneous with respect to genetic background, body fat distribution, cardiometabolic risk burden, and dementia epidemiology, and typically involve shorter follow-up durations and younger baseline populations.^15^ Additionally, the United States has a more racially, ethnically, and socioeconomically diverse population than many Asian countries.^14^, ^15^, ^16^

Our study meaningfully extends this literature by demonstrating that the excess neurodegenerative risk associated with nonobese MASLD is not confined to East Asian populations. Using NHANES III, we capture a racially, ethnically, and socioeconomically diverse US population, including African American, Mexican American, White and other groups with substantially longer follow-up, allowing for robust ascertainment of AD mortality into advanced age. Importantly, the magnitude and consistency of associations observed in this US cohort—despite a lower overall prevalence of lean MASLD—suggest that the phenotype confers intrinsic vulnerability that transcends regional, ethnic, and health-care system differences, as well as more racial and ethnic.

### Potential Biological Mechanisms

Several mechanisms may plausibly explain the heightened AD mortality observed among individuals with nonobese MASLD. Unlike obese MASLD, which is often driven by generalized adiposity, nonobese MASLD may reflect disproportionate visceral adiposity and ectopic fat deposition despite normal or near-normal BMI.^17^ This pattern is associated with heightened hepatic and systemic inflammation, oxidative stress, and insulin resistance—pathways increasingly implicated in neurodegeneration.^18^

Additionally, lean and nonobese MASLD have been linked to greater fibrosis severity relative to BMI, raising the possibility that fibrosis-related inflammatory signaling contributes to accelerated cognitive decline.^19^ Sarcopenia and altered muscle–liver crosstalk, which are more common in lean MASLD, may further exacerbate metabolic dysregulation and brain insulin resistance. Genetic susceptibility, including variants affecting lipid handling and hepatic inflammation, may also play a larger role in nonobese MASLD and could partially account for the observed associations.^20^

### Why Effects May Appear Stronger in the US Context

Interestingly, the relative risk estimates observed in our US cohort are comparable to, and in some cases exceed, those reported in East Asian studies. This may reflect differences in competing risks, background dementia incidence, and survival patterns. East Asian populations generally exhibit lower baseline dementia rates and lower cardiometabolic mortality, potentially attenuating observable associations.^21^ In contrast, the higher burden of cardiometabolic disease, vascular risk factors, and longevity in the US may unmask the long-term neurodegenerative consequences of nonobese MASLD.^22^

### Clinical and Public Health Implications

Our findings carry important implications for risk stratification and screening. Current clinical paradigms often emphasize obesity as the primary driver of MASLD-related complications, potentially leading to underrecognition of nonobese disease. The elevated AD mortality risk observed in nonobese MASLD underscores the need for clinicians to consider hepatic steatosis as a marker of systemic metabolic vulnerability regardless of body size. From a public health perspective, these results support broader metabolic and cognitive surveillance strategies that extend beyond traditional BMI-based risk assessment.

## Conclusion

In this large, nationally representative US cohort, nonobese MASLD emerged as a distinct metabolic phenotype associated with significantly higher AD mortality, independent of traditional risk factors. These findings extend prior East Asian observations to a heterogeneous Western population and underscore the need to move beyond BMI-centric frameworks when assessing MASLD-related risk. Future studies should focus on elucidating underlying mechanisms, refining phenotype-based risk stratification, and evaluating targeted interventions to mitigate long-term cognitive decline in this overlooked population.
